# CDKN2a/p16 Antagonizes Hepatic Stellate Cell Activation and Liver Fibrosis by Modulating ROS Levels

**DOI:** 10.3389/fcell.2020.00176

**Published:** 2020-03-24

**Authors:** Fangqiao Lv, Nan Li, Ming Kong, Jun Wu, Zhiwen Fan, Dengshun Miao, Yong Xu, Qing Ye, Yutong Wang

**Affiliations:** ^1^Department of Cell Biology, Municipal Laboratory for Liver Protection and Regulation of Regeneration, School of Basic Medical Sciences, Capital Medical University, Beijing, China; ^2^Key Laboratory of Targeted Intervention of Cardiovascular Disease and Collaborative Innovation Center for Cardiovascular Translational Medicine, Department of Pathophysiology, Nanjing Medical University, Nanjing, China; ^3^Department of Anatomy, Nanjing Medical University, Nanjing, China; ^4^Department of Pathology, The Affiliated Drum Tower Hospital, Nanjing University Medical School, Nanjing, China; ^5^Institute of Biomedical Research, Liaocheng University, Liaocheng, China

**Keywords:** CDKN2a/p16, liver fibrosis, ROS, p38 MAPK, hepatic stellate cell

## Abstract

The lipid-storage hepatic stellate cells (HSC) play as pivotal role in liver fibrosis being able to *trans-*differentiate into myofibroblasts in response to various pro-fibrogenic stimuli. In the present study we investigated the role of CDKN2a/p16, a negative regulator of cell cycling, in HSC activation and the underlying mechanism. Levels of p16 were significantly down-regulated in activated HSCs isolated from mice induced to develop liver fibrosis compared to quiescent HSCs isolated from the control mice *ex vivo*. There was a similar decrease in p16 expression in cultured HSCs undergoing spontaneous activation or exposed to TGF-β treatment *in vitro.* More important, p16 down-regulation was observed to correlate with cirrhosis in humans. In a classic model of carbon tetrachloride (CCl_4_) induced liver fibrosis, fibrogenesis was far more extensive in mice with p16 deficiency (KO) than the wild type (WT) littermates. Depletion of p16 in cultured HSCs promoted the synthesis of extracellular matrix (ECM) proteins. Mechanistically, p16 deficiency accelerated reactive oxygen species (ROS) generation in HSCs likely through the p38 MAPK signaling. P38 inhibition or ROS cleansing attenuated ECM production in p16 deficient HSCs. Taken together, our data unveil a previously unappreciated role for p16 in the regulation of HSC activation. Screening for small-molecule compounds that can boost p16 activity may yield novel therapeutic strategies against liver fibrosis.

## Introduction

Liver fibrosis is part of a host defense response taking place when the liver is exposed to a host of injuries such as corrosive chemicals, toxins, pathogens, ischemia, excessive nutrients, and certain medications (Friedman, 2010; Hernandez-Gea and Friedman, 2011; Luedde and Schwabe, 2011; Manmadhan and Ehmer, 2019; Spinnenhirn et al., 2019). Being a tightly regulated pathophysiological process, liver fibrosis contributes to the wound healing and repair of the injured liver parenchyma thus protecting the architectural and functional integrity of the liver. On the contrary, uncontrolled and aberrant liver fibrosis disrupts the normal hepatic structure and heralds such end-stage liver diseases as hepatocellular carcinoma (HCC) and cirrhosis (Seki and Schwabe, 2015). Decades of rigorous research have uncovered myofibroblast, a specialized cell type that possesses both a contractile phenotype (muscle-like) and the ability to produce and secrete extracellular matrix proteins (fibroblast-like), as the key mediator of liver fibrosis (Kisseleva, 2017). Although various types of cells, including the liver resident cells and circulating cells, have been proposed as the potential source of myofibroblasts during liver fibrosis, lineage tracing/fate-mapping experiments performed in experimental animals have unequivocally demonstrated that hepatic stellate cells (HSCs), the lipid-storage cells tucked between the liver parenchyma and the sinusoidal endothelium, are the predominant origin from which myofibroblasts are derived from Mederacke et al. (2013).

A host of factors contribute to the *trans-*differentiation of HSCs to mature myofibroblasts, including transforming growth factor (TGF-β), platelet derived growth factor (PDGF-BB), high glucose, and reactive oxygen species (ROS) (Liu and Desai, 2015; Alzahrani et al., 2018; Hou and Syn, 2018; Jia et al., 2018). ROS production, similar to fibrogenesis, is also considered a host defense mechanism (Sies, 2015). Mounting evidence points to a link between excessive ROS accumulation and HSC activation. For instance, mice with deficiencies in one of the enzymes involved in the catalysis of ROS generation, including NADPH oxidase 1 (NOX1), NOX2, and NOX4, exhibit weaker liver fibrogenic response with attenuated HSC activation (Paik et al., 2011; Bettaieb et al., 2015; Lan et al., 2015). In contrast, deletion of Nrf2, the master regulator of anti-oxidative stress, leads to aggravated liver fibrosis in mice (Xu et al., 2008). In accordance, administration of antioxidants has been shown to alleviate liver fibrosis in experimental animals (Cho and Song, 2018; Li et al., 2018d; Chen et al., 2019). Among the myriad signaling pathways that regulate ROS production, the mitogen activated protein kinase (MAPK) pathway has been shown to play a key role. Hattori et al. have reported that a specific p38/MAPK inhibitor FR-167653 dampens HSC activation in cirrhotic rats (Hattori et al., 2007). Kluwe et al. (2010) have reported that mice deficient in JNK1/MAPK or treated with the pan-JNK inhibitor SP600125 are protected from liver fibrosis induced by CCl_4_ injection or bile duct ligation (BDL).

P16, encoded by CDKN2a, is a negative regulator of cell cycling (Rayess et al., 2012). We have previously shown that p16 knockout mice, when placed on a pro-steatotic diet, exhibit more severe lipid deposition, inflammatory infiltration, and ROS accumulation in the liver compared to the WT mice (Lv et al., 2017). Here we report that p16 deficiency amplifies liver fibrosis in mice by enhancing HSC activation. Mechanistically, p16 deficiency activates p38/MAPK signaling to promote ROS production in HSCs. Therefore, out data provide a strong rationale for the screening of small-molecule compounds that can boost p16 activity in the therapeutic intervention of liver fibrosis.

## Materials and Methods

### Animals

Male BABL/c mice with p16 KO (p16^–/–^), ca. 6–8 weeks old, were used in these studies. To induce liver fibrosis, carbon tetrachloride (CCl_4_, 1.0 mL/kg as 50% vol/vol) or corn oil is administered to mice every other day for 7 days. Then the mice were sacrificed 24 h after the last injection and the liver samples were used for further analysis. Alternatively, liver fibrosis was induced by bile duct ligation (BDL) as previously described (Kong et al., 2019b). Briefly, the mice were anesthetized with inhalation of 4% isoflurane. The abdomen was open to expose the bile duct. A 5–0 suture was placed around the bile duct and two surgical knots were tied to ensure effective obstruction. The mice were sacrificed 2 weeks after the procedure. Plasma ALT/AST and liver hydroxyproline levels were determined with kits according to the manufacturer’s protocol (Jiancheng Bioengineering, China). All animal experiments were approved by NJMU Intramural Ethics Committee on Animal Studies.

### Cell Culture

Immortalized human HSC cells (LX-2, ATCC) were maintained in Dulbecco’s modified Eagle’s medium (DMEM; GibcoTM, Thermo Fisher Scientific, Waltham, MA, United States) supplemented with 10% fetal bovine serum (FBS; ExCell Bio, Genetimes, Shanghai, China) as previously described (Kong et al., 2019a). Cells were used typically between 3rd and 5th passages. Cells were plated at the density of 3 × 10^5^ cells/well in 6-well culture dishes. Primary stellate cells were isolated by collagenase P digestion and Density gradient centrifugation as previously described (Lu et al., 2019). Small interfering RNA (siRNA) targeting human Cdkn2a (5′-ACACCGCUUCUGCCUUUUCTT-3′) and non-silencing RNA were transfected at final concentration of each 50 nM into LX-2 cells using a Lipofectamine RNAi MAX kit (Thermo Fisher Scientific) according to the manufacturer’s instructions. EdU analysis was performed using Cell-Light EdU Apollo643 *in vitro* Kit according the manufacturer’s instruction (RiboBio, Guangzhou, China). CCK-8 analysis was performed using Cell Counting Kit-8 according the manufacturer’s instruction (Dojindo, Japan). TGF-β was purchased from PeproTech. Prior to TGF-β treatment, LX-2 cells were serum starved overnight. The next day, TGF-β was diluted in serum-free media to treat the cells.

### Histochemical and Immunofluorescence Staining

The specimens were collected after sacrificing the mice. Then they were fixed in 10% formalin and embedded in paraffin. Paraffin tissue sections were stained with Masson’s trichrome or Sirius Red for histomorphometric assessment. The liver samples of cirrhotic patients in the form of tissue array (Alenabio, China) were collected from Tongxu County People’s Hospital, China. The study was approved by the Ethics Committee of Tongxu County People’s Hospital. The length of the biopsy specimen is around 2 mm. 5 cirrhosis samples and 5 healthy liver samples used as control were included in the present study. The patients’ information is summarized in Supplementary Table S1. Sections were deparaffinized and dehydrated. For antigen retrieval, they were heating in a microwave in EDTA repairing buffer (pH 9.0) for 15 min followed by blocking with 5% BSA. The sections were incubated with anti-p16 (1:200, Proteintech, catalog# 10883-1) and anti-Desmin (1:200, Thermo Fisher Scientific, United States, catalog# MA5-13259) overnight at 4°C. After briefly washing, the sections were incubated with goat anti-rabbit IgG and goat anti-mouse IgG secondary antibodies and DAPI.

### Western Blot

The protein was extracted from total cells or tissues using RIPA buffer containing PMSF and phosphatase inhibitor as previously described (Li et al., 2019a,b,c,d,e; Liu et al., 2019a, b; Lu et al., 2019; Shao et al., 2019; Weng et al., 2019; Yang et al., 2019a, b; Zhao et al., 2019). 30 μg of protein were loaded in each lane and separated by 8% PAGE-SDS gel with all-blue protein markers (Bio-Rad). Proteins were transferred to nitrocellulose membranes (Bio-Rad) in a Mini-*Trans-*Blot Cell (Bio-Rad). The membranes were blocked with 5% fat-free milk powder in Tris-buffered saline at room temperature for half an hour and then incubated with the following primary antibodies at 4°C overnight: Western blot analyses were performed with anti-p16 (1:1,000, Proteintech, catalog# 10883-1), anti-Collagen type I (1:1,000, Proteintech, catalog# 14695-1), anti-α-SMA (1:5,000, Abcam, catalog# ab5694), anti-GAPDH (1:2,000, Proteintech, catalog# 60004-1), anti-p38 (1:1,000, Proteintech, catalog# 14064-1), and anti-phospho-p38 antibodies (1:2,000, Cell Signaling, catalog# 1682). Image J software was used for densitometrical quantification and densities of target proteins were normalized to those of β-actin. Data are expressed as relative protein levels compared to the control group which is arbitrarily set as 1.

### RNA Isolation and Real-Time RT-PCR

Total RNA was extracted with Trizol reagent (Sigma, United States) from cells or tissues stored in a −80°C freezer. Reverse transcriptase reactions were performed with 1 μg of RNA using Reverse Transcription System (Promega, United States) as previously described (Fan et al., 2017, 2019; Li et al., 2017, 2018b,c,e,f,g; Yu et al., 2017; Liu et al., 2018; Yang et al., 2018; Zeng et al., 2018; Zhang et al., 2018; Kong et al., 2019a, b). Real-time RT-PCR was performed with TransStart Green qPCR SuperMix (TransGen Biotech, Beijing, China). Primers were as follow: mouse p16: forward 5′-GGGTTTTCTTGGTGAAGTTCG-3′ and reverse 5′-TTGCCCATCATCATCACCT-3′; mouse Col I: forward 5′- GACGCCATCAAGGTCTACTG-3′ and reverse 5′- ACGGGAATCCA-TCGGTCA-3′; mouse α-SMA: forward 5′- CTGAGCGTGGCTATTCCTTC-3′ and reverse 5′- CTTCTGCATCCTGTCAGCAA-3′; human p16: forward 5′-CGAATAGTTACGGTCGGAGG-3′ and reverse 5′-TGAGAGTGGCGGGGTCG-3′; human Col I: forward 5′- AGGCGAACAGGGCGACAGAG-3′ and reverse 5′- GGCCAGGGAGACCGTTGAGT-3′; human α-SMA: forward 5′- CATCCTCCCTTGAGAAGAGTTA-3′ and reverse 5′- TACATAGTGGTGCCCCCTGATA-3′; Ct values of target genes were normalized to the Ct values of housekeeping control gene (18s, 5′-CGCGGTTCTATTTTGTTGGT-3′ and 5′-TCGTCTTCGAAACTCCGACT-3′ for both human and mouse genes) using the ΔΔCt method and expressed as relative mRNA expression levels compared to the control group which is arbitrarily set as 1.

### ROS Measurement

LX-2 cells were cultured in 96-well plates and transfected with N.C. or siCDKN2A. 24 h after transfection, 2′,7′-dichlorodihydrofluorescein (DCF) was added to and incubated with the cells at 37°C for 30 min. Frozen tissue sections were treated with autofluorescence quench for 5 min. After brief washing, the sections were incubated with the staining solution at 37°C for 30 min. After 3 washes with PBS, the cells or the sections were visualized with a fluorescence microscope (NIKON ECLIPSE C1).

### Statistical Analysis

The data were calculated and expressed as mean SEM. Student *t* tests were performed with GraphPad Prism. *p* values smaller than 0.05 were considered significant.

## Results

### P16 Down-Regulation in HSCs Correlates With Liver Fibrogenesis

**FIGURE 1:**
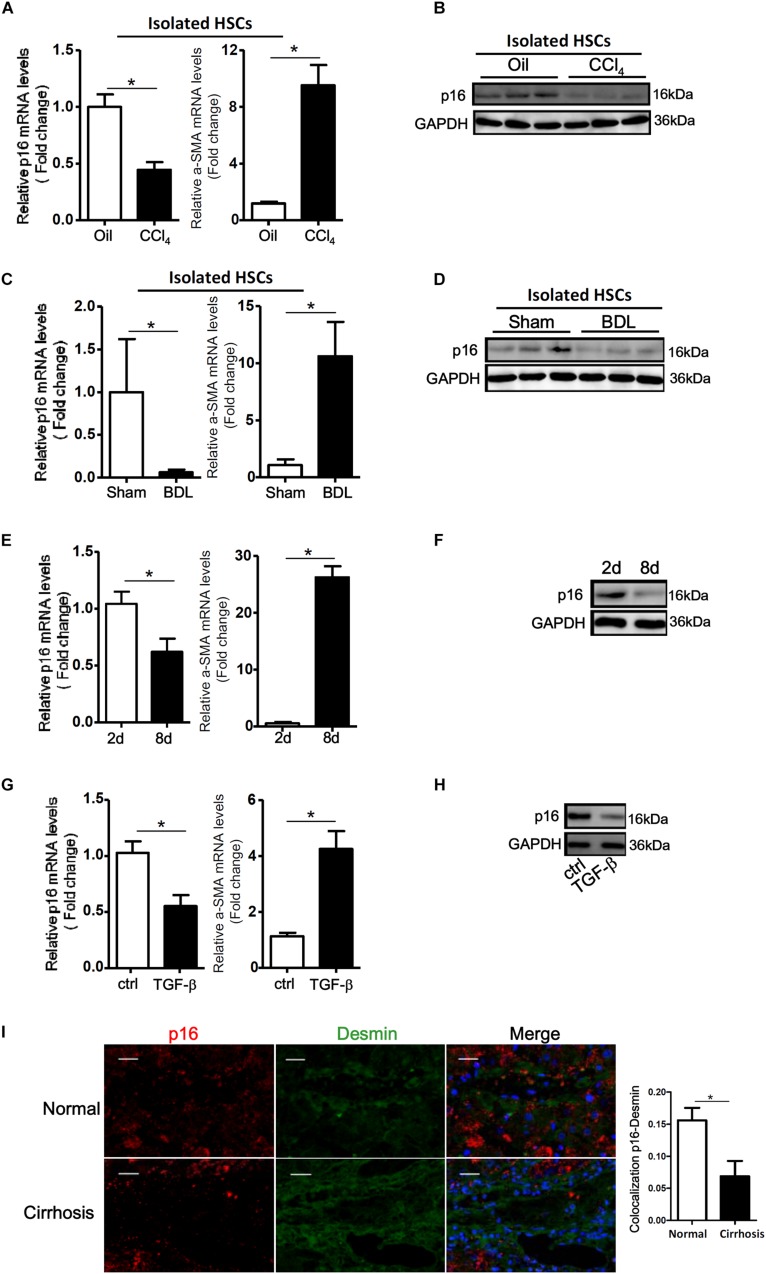
p16 expression is down-regulated during HSC activation. **(A,B)** C57/BL6 mice were induced to develop liver fibrosis by CCl_4_ injection and primary HSCs were isolated as described in Methods. P16 expression was examined by qPCR and Western. **(C,D)** C57/BL6 mice were induced to develop liver fibrosis by BDL and primary HSCs were isolated as described in Methods. P16 expression was examined by qPCR and Western. **(E,F)** Primary mouse HSCs were isolated from C57/BL6 mice and allowed to undergo spontaneous activation. The cells were harvested at day 2 and day 8 and p16 expression was examined by qPCR and Western. **(G,H)** LX-2 cells were treated with or without TGF-β (10 ng/ml) for 48 h. P16 expression was examined by qPCR and Western. Data represent averages of three independent experiments and error bars represent SEM. *p<0.05. **(I)** Normal and cirrhotic liver biopsy specimens were stained with anti-p16 and anti-desmin. *N* = 5 for each group. Scale bar, 200 μm. *p<0.05.

We first tackled the question as to whether p16 levels would be altered during HSC activation. To this end, we used a series of different cell and animal models. In the first model wherein C57/BL6 mice were injected with CCl_4_ every other day for a week, p16 levels were significantly down-regulated by 53% in the freshly isolated HSCs, accompanying an increase in α-SMA levels, compared to those isolated from mice injected with corn oil (Figures 1A,B). In a second model in which the mice were subjected to bile duct ligation (BDL) to induce liver fibrosis, we again found that p16 levels were decreased by more than 85% in freshly isolated HSCs 2 weeks after the surgical procedure compared to the those isolated from the sham mice (Figures 1C,D). QPCR performed using whole liver homogenates revealed similar results showing a down-regulation of p16 and an up-regulation of α-SMA (Supplementary Figure S1). Of interest, expression levels of check point kinase 1 (Chk1), a regulator of cell cycle, was not significantly altered in either isolated HSCs or in liver homogenates (Supplementary Figure S2).

We next isolated primary HSCs from normal C57/BL6 mice and allowed these cells to undergo spontaneous activation *in vitro*. As shown in Figures 1E,F, activated HSCs (8d) exhibited lower p16 expression levels than quiescent HSCs (2d). We also treated LX-2, an immortalized human HSC strain, with the pro-fibrogenic growth factor TGF-β. Exposure of LX-2 cells to TGF-β similarly reduced p16 levels (Figures 1G,H). Of note, TGF-β treatment down-regulated p16 expression in LX-2 cells in a dose-dependent manner (Supplementary Figure S3).

Finally, we examined the levels of p16 in patients with cirrhosis. Compared to the healthy livers, p16 expression was significantly down-regulated in HSCs (identified by desmin) in cirrhotic livers (Figure 1). Collectively, these data suggest that there might be a correlation between p16 down-regulation and liver fibrogenesis both *in vivo* and *in vitro*.

### P16 Deficiency Promotes Liver Fibrogenesis in Mice

**FIGURE 2:**
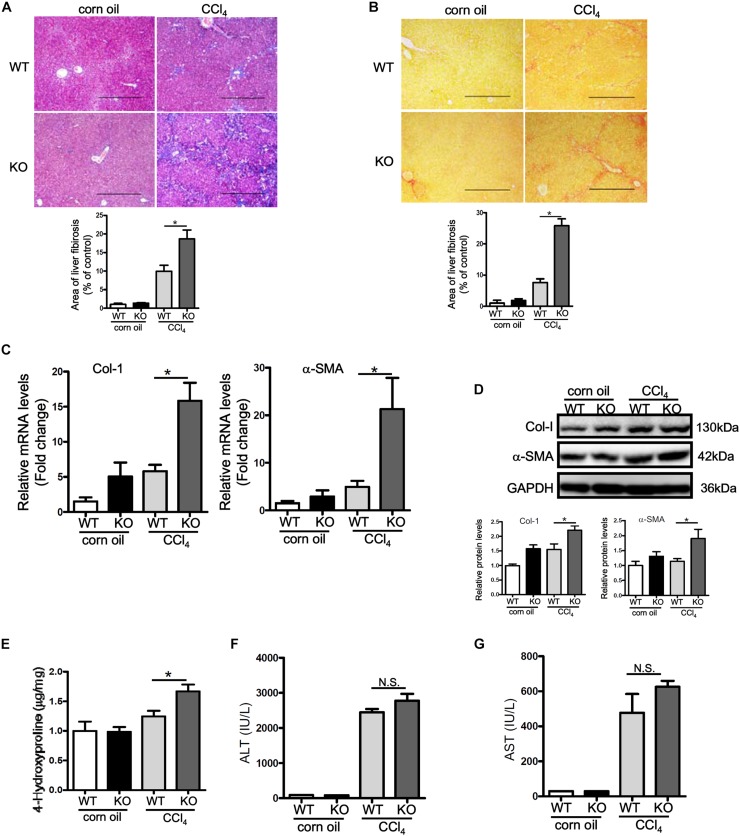
p16 deficiency exacerbates liver fibrosis in mice. P16 knockout (KO) and wild type (WT) mice were injected with CCl_4_ to induce liver fibrosis as described in Methods. **(A)** Masson’s trichrome staining. **(B)** Picrosirius red staining. **(C,D)** Expression of pro-fibrogenic genes was examined by qPCR and Western. **(E)** Hepatic hydroxyproline levels. **(F)** Plasma ALT levels. **(G)** Plasma AST levels. *N* = 5 mice for each group. *p < 0.05.

Having observed that p16 down-regulation accompanies liver fibrogenesis, we hypothesized that p16 deletion in mice might accelerate the fibrogenic response. P16 deficient (KO) mice and their wild type (WT) littermates were injected with CCl_4_ to induced liver fibrosis. Masson’s trichrome staining (Figure 2A) and picrosirius red staining (Figure 2B) revealed that deposition of ECM proteins was far more extensive in KO mice than in WT mice. Relative quantitative PCR (Figure 2C) and Western blotting (Figure 2D) confirmed that expression levels of pro-fibrogenic genes, including collagen type I and α-SMA, were higher in KO mice compared to WT mice. Relative quantitative PCR revealed that levels of interleukin 1 (Il-1) and interleukin 6 (Il-6) were higher in the KO livers than in the WT livers whereas levels of macrophage chemoattractant protein 1 (Mcp1) and tumor necrosis factor (Tnf) were comparable in the KO livers and in the WT livers (Supplementary Figure S4). Additional evidence to support p16 deficiency promoting liver fibrogenesis was provided by hepatic hydroxyl proline (HHP) quantification assay, which showed that increased HHP levels in KO mice than in WT mice (Figure 2E). Of interest, p16 deficiency appeared to have little impact on liver injury as evidenced by the observation that KO mice and WT mice exhibit comparable plasma ALT (Figure 2F) and AST (Figure 2G) levels, suggesting that the changes in liver fibrogenesis could be attributed to a HSC-autonomous role for p16.

### P16 Deficiency Accelerates HSC Activation *in vitro*

**FIGURE 3:**
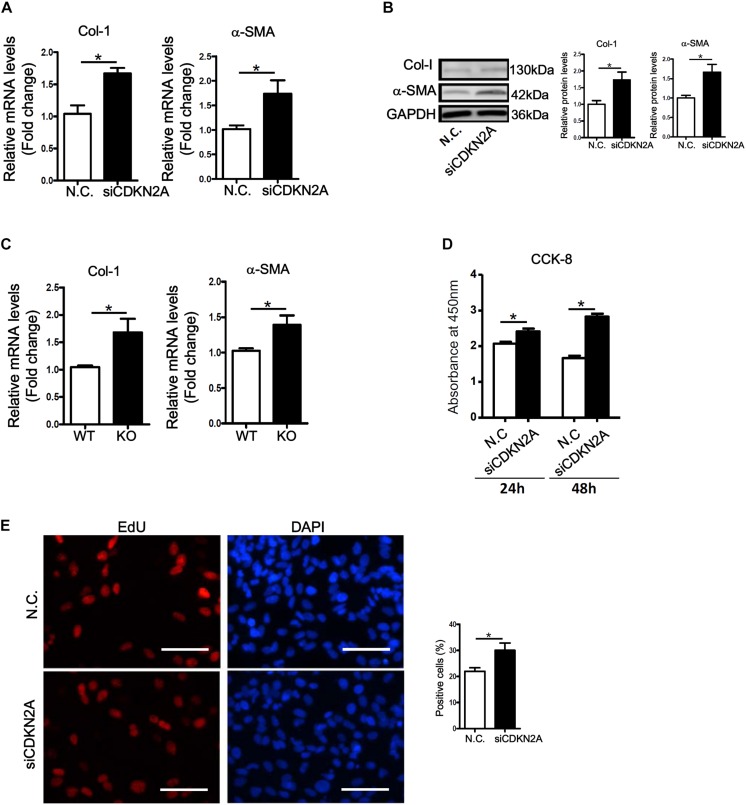
p16 deficiency accelerates HSC activation. **(A,B)** LX-2 cells were transfected with siRNA targeting p16 or scrambled siRNA (SCR). Expression of pro-fibrogenic genes was examined by qPCR and Western. **(C)** Primary HSCs were isolated from p16 knockout (KO) and wild type (WT) mice and allowed to undergo spontaneous activation for 8 days. Expression of pro-fibrogenic genes was examined by qPCR. **(D,E)** LX-2 cells were transfected with siRNA targeting p16 (50 nM) or scrambled siRNA (SCR). Cell proliferation was measured by CCK-8 activity and EdU incorporation as described in Methods. Scale bar, 50 μm. Data represent averages of three independent experiments and error bars represent SEM. *p<0.05.

We next examined whether p16 deficiency in HSCs would be sufficient to stimulate its activation. Small interfering RNA (siRNA) mediated knockdown of p16 significantly up-regulated the expression of collagen type I and α-SMA at both mRNA (Figure 3A) and protein (Figure 3B) levels in LX-2 cells. ELISA assay confirmed secreted collagen levels were also up-regulated following p16 depletion (Supplementary Figure S5). In addition, activation of primary HSCs isolated from p16 KO mice was faster than those isolated from WT mice as evidenced by higher expression levels of collagen type I and α-SMA (Figure 3C). HSC activation is characterized by, in addition to robust production of ECM proteins, enhanced proliferation. P16 knockdown significantly promoted proliferation of LX-2 cells as measured by CCK-8 levels (Figure 3D) and BrdU incorporation rate (Figure 3E).

### P16 Deficiency Promotes ROS Production During HSC Activation

**FIGURE 4:**
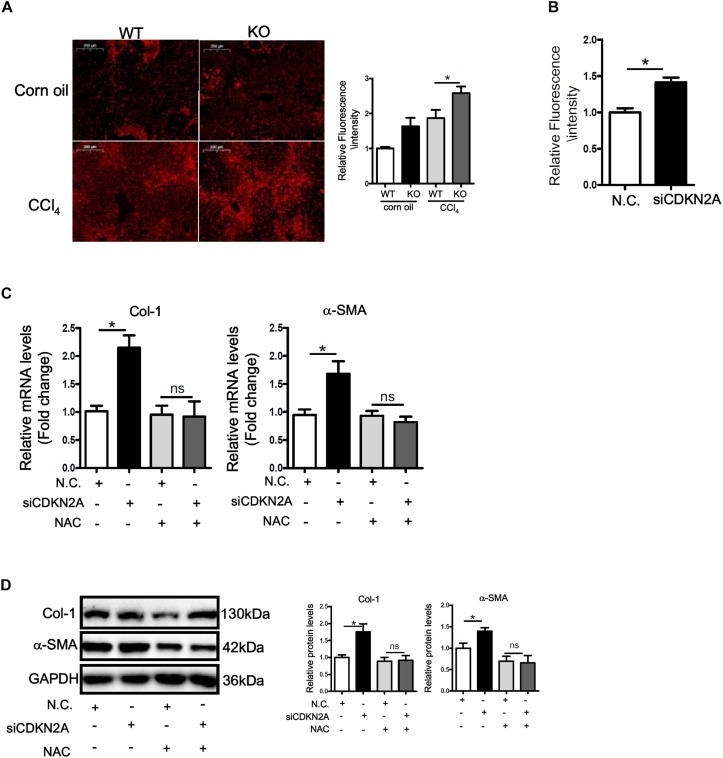
p16 deficiency accelerates HSC activation by promoting ROS production. **(A)** P16 knockout (KO) and wild type (WT) mice were injected with CCl_4_ to induce liver fibrosis as described in Methods. ROS levels were examined by DHE staining and quantified by Image Pro. Scale bar, 200 μm. **(B)** LX-2 cells were transfected with siRNA targeting p16 (50 nM) or scrambled siRNA (SCR). Measurement of ROS production was tested by DHE staining and quantified by Image Pro. **(C,D)** LX-2 cells were transfected with siRNA targeting p16 or scrambled siRNA (SCR) followed by treatment with NAC. Expression of pro-fibrogenic genes was examined by qPCR and Western. Data represent averages of three independent experiments and error bars represent SEM. *p<0.05.

ROS accumulation plays a key role in HSC activation and liver fibrosis. We asked whether accelerated HSC activation as a result of p16 deficiency could be accounted for by ROS accumulation. DHE staining showed that ROS levels were significantly higher in the livers of CCl_4_-injected mice than in the control livers; p16 deletion further up-regulated ROS levels in the fibrotic livers (Figure 4A). Similarly, p16 knockdown in LX-2 cells also increased ROS levels (Figure 4B). Next, we attempted to remove intracellular ROS by treating the cells with the antioxidant n-acetylcysteine (NAC). As shown Figures 4C,D, NAC treatment completely blunted the pro-fibrogenic effect of p16 depletion by normalizing the mRNA and protein levels of collagen type I and α-SMA. Similar observation was made with regard to secreted collagen levels (Supplementary Figure S6).

### P38 MAPK Mediates ROS Production During HSC Activation

**FIGURE 5:**
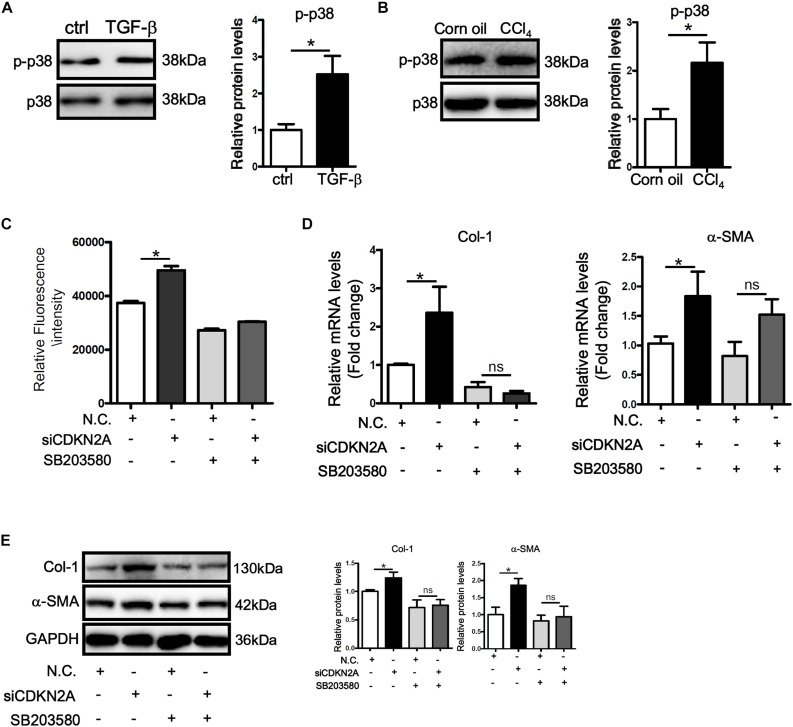
p16 regulates HSC activation by modulating p38 signaling. **(A)** LX-2 cells were treated with or without TGF-β (2 ng/ml). Phosphorylation levels of p38 were examined by Western. **(B)** C57/BL6 mice were induced to develop liver fibrosis by CCl_4_ injection as described in Methods. Phosphorylation levels of p38 were examined by Western. **(C–E)** LX-2 cells were transfected with siRNA targeting p16 (50 nM) or scrambled siRNA (SCR) followed by treatment with SB203580. ROS levels were examined by DHE staining and quantified by Image Pro. Expression of pro-fibrogenic genes was examined by qPCR and Western. Data represent averages of three independent experiments and error bars represent SEM. *p<0.05.

MAPK signaling plays a critical role in ROS production (Son et al., 2011). Western blotting showed that activity of p38 MAPK, as judged by its phosphorylation status, was up-regulated by TGF-β treatment in LX-2 cells (Figure 5A). Similarly, p38 activity was higher in the fibrotic livers in CCl_4_-injected mice than in the control livers (Figure 5B). In accordance, treatment with a specific p38 inhibitor (SB203580) not only attenuated basal ROS levels in LX-2 cells but abrogated TGF-β induced ROS production (Figure 5C). P38 inhibition also prevented the augmentation of collagen type I and α-SMA following p16 knockdown at both mRNA (Figure 5D) and protein (Figure 5E) levels. Secreted collagen levels exhibited similar patterns as evaluated by ELISA (Supplementary Figure S7).

## Discussion

ROS-mediated activation of HSCs represents a paradigm in the pathogenesis of liver fibrosis. This notion is buttressed by a plethora of evidence (Paik et al., 2014; Crosas-Molist and Fabregat, 2015). Here we describe a pathway in which the cell cycling inhibitor CDKN2a/p16 regulates ROS production and HSC activation by modulating p38 activity. One of the better characterized functions of p16 is the regulation of cell cycling in which it acts to keep the cells in a quiescent state (Ruas and Peters, 1998). In the normal livers, HSCs are considered in a non-proliferative state having temporarily exited the cell cycle (Rippe and Brenner, 2004). Therefore, it makes sense that down-regulation of p16 expression during HSC activation may serve as the permissive step for the cells to exit the G0 phase. In addition to p16, there are other factors that can potentially antagonize liver fibrosis by forcing a quiescent state on the HSCs. For instance, statins exert their hepato-protective effects by inducing the transcription factor KLF2, which in turn promotes NO production in liver sinusoidal endothelial cells. NO, via a paracrine mechanism, induces a quiescent phenotype in HSCs (Marrone et al., 2013). Sun et al. (2016) have reported that a SUMOylation dependent pathway represses SIRT1 transcription in quiescent HSCs, which allows the cells to resume proliferation thereby leading to liver fibrosis. Several independent reports have portrayed the nuclear receptor PPARγ as a key regulator of HSC quiescence, either by inhibiting the pro-fibrotic JunD activity (Hazra et al., 2004), by inducing an adipogenic *trans-*differentiation program (She et al., 2005), or by suppressing ROS production (Hsu et al., 2013). Coincidently, both PPARγ and KLF2 have been shown, under various circumstances, to activate p16 expression (Guan et al., 1999; Wang et al., 2017). Because expression levels and/or activities of PPARγ and KLF2 are down-regulated during HSC activation (Marrone et al., 2015; Li et al., 2018a), it is reasonable to postulate that PPARγ and KLF2 may contribute to suppression of HSC activation by restoring p16 expression. Our data thus suggest that p16 might be a key node in the intertwined network that regulates HSC phenotype and are consistent with the theme that maintenance of HSC quiescence is an attractive path through which antifibrotic strategies should be devised.

One intriguing question is whether ROS production is coupled to the regulation of cell cycling and, if so, whether p16 is moderator of these two inter-related processes. Indeed, mounting evidence suggests that cell cycling is programmed, at least in part, by the Redox status. It has been proposed that ROS-sensitive κ of NF-κB probably provides the necessary cue for quiescent HSCs to re-enter the proliferative cycle (Sarsour et al., 2009). Of note, Ghiorzo et al. (2004) have shown that there is an inverse correlation between p16 expression and NF-κB expression in melanoma tissues, suggesting that p16 down-regulation may be permissive in ROS accumulation and consequently HSC activation. This hypothesis can find support in our observation that inhibition of p38, which is upstream of and required for the non-canonical activation of NF-κB (Cristofanon et al., 2009), attenuated ROS accumulation and HSC activation. However, there is also evidence to suggest that NF-κB activates p16 expression (Chien et al., 2011). In addition, Jenkins et al. have reported that the ability of p16 to suppress ROS production is independent the cell cycling status (Jenkins et al., 2011). Therefore, more investigations should be conducted to reconcile these conflicting reports.

Hepatic stellate cell activation is not a strictly cell-autonomous process. In other words, non-HSCs including both intra- and extra-hepatic cells may influence the HSC phenotype. Since we have used a global CDKN2a knockout mouse strain in the present study, the possibility that p16 may exert cell-specific roles in regulating liver fibrosis cannot be ignored. For instance, p16 is known to suppress the production of pro-inflammatory cytokines in macrophages by promoting IRAK1 degradation (Murakami et al., 2012). Since pro-inflammatory mediators serve as a driving force for HSC activation, it is conceivable that the observed amelioration of liver fibrosis as a result of p16 deficiency could be attributed to a defect in macrophage-mediated inflammation. Similarly, hepatocyte death is considered a key factor promoting HSC activation (Brenner et al., 2013). p16 activation by β-catenin has been shown to protect hepatocytes from cell death, which may limit HSC activation (Fischer et al., 2007). Cell-specific p16 transgenic animal models may provide additional insights on the mechanism(s) by which p16 contributes to HSC activation and liver fibrosis.

## Conclusion

In conclusion, our data point to a novel role for p16 as a suppressor of HSC *trans-*differentiation and liver fibrosis. The major limitations of the present study include the reliance on a single animal model, instead of multiple models, and an immortalized cell line (LX-2), as opposed to primary HSCs. Further studies are warranted to determine whether boosting p16 activity can be considered as a reasonable approach in the intervention of liver fibrosis.

## Data Availability Statement

The raw data supporting the conclusions of this article will be made available by the authors, without undue reservation, to any qualified researcher.

## Ethics Statement

The studies involving human participants were reviewed and approved by the Capital Medical University Ethics Committee on Human Studies. The patients/participants provided their written informed consent to participate in this study. The animal study was reviewed and approved by Nanjing Medical University Ethics Committee on Humane Treatment of Experimental Animals.

## Author Contributions

YX and FL conceived the project and wrote the manuscript. FL, NL, MK, and YX designed the experiments. FL, NL, MK, JW, and ZF performed the experiments and collected the data. YW and QY secured funding. YW and QY provided supervision. DM provided key reagents.

## Conflict of Interest

The authors declare that the research was conducted in the absence of any commercial or financial relationships that could be construed as a potential conflict of interest.

## Abbreviations

ECM: extracellular matrix
HSC: hepatic stellate cell
MAPK: mitogen activated protein kinase
ROS: reactive oxygen species
TGF: transforming growth factor.
