# Supplementary material for: CDKN2a/p16 Antagonizes Hepatic Stellate Cell Activation and Liver Fibrosis by Modulating ROS Levels
Source: Front Cell Dev Biol. 2020 Mar 24;8:176. doi: 10.3389/fcell.2020.00176 (PMC7105638; doi:10.3389/fcell.2020.00176)
Supplement: Supplementary file 1 [file Data_Sheet_1.pdf]

Table I: The human liver samples were acquired from Henan Tongxu People's Hospital. The study was approved by the Ethics Committee of Henan Tongxu People's Hospital. The human liver samples including 5 cirrhosis liver samples and 5 healthy liver samples were used as control. The metabolic index are not available, the patient's other information are as follows:

	age	sex	pathological diagnosis
Normal	35	M	Hepatic tissue
	21	F	Hepatic tissue with fatty degeneration
	43	M	Hepatic tissue
	40	M	Hepatic tissue
	45	M	Hepatic tissue
Cirrhosis	60	M	Nodular cirrhosis
	57	M	Nodular cirrhosis
	55	M	Nodular cirrhosis
	51	M	Nodular cirrhosis
	55	M	Nodular cirrhosis

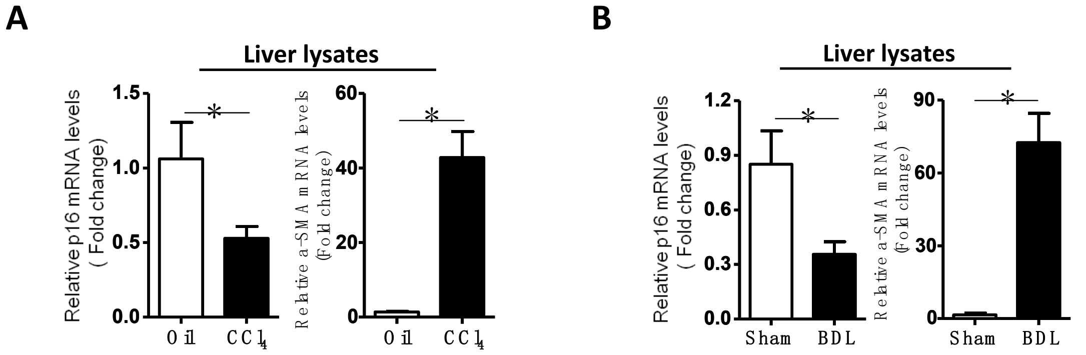

Fig.S1: (**A**) C57/BL6 mice were induced to develop liver fibrosis by CCl_4 injection as described in Methods. P16 expression in whole liver homogenates was examined by qPCR. (**B**) C57/BL6 mice were induced to develop liver fibrosis by BDL as described in Methods. P16 expression in whole liver homogenates was examined by qPCR.

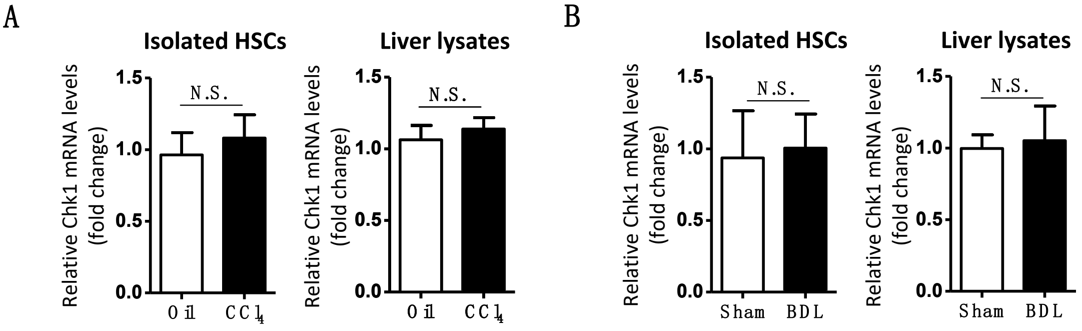

Fig.S2: (**A**) C57/BL6 mice were induced to develop liver fibrosis by CCl_4 injection as described in Methods. Chk1 expression in isolated HSCs And in whole liver homogenates was examined by qPCR. (**B**) C57/BL6 mice were induced to develop liver fibrosis by BDL as described in Methods. Chk1 expression in isolated HSCs And in whole liver homogenates was examined by qPCR.

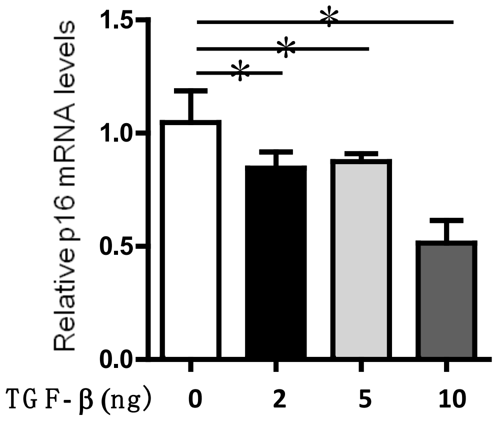

Fig.S3: LX-2 cells were treated with different concentrations of TGF-β for 24h. p16 expression was examined by qPCR.

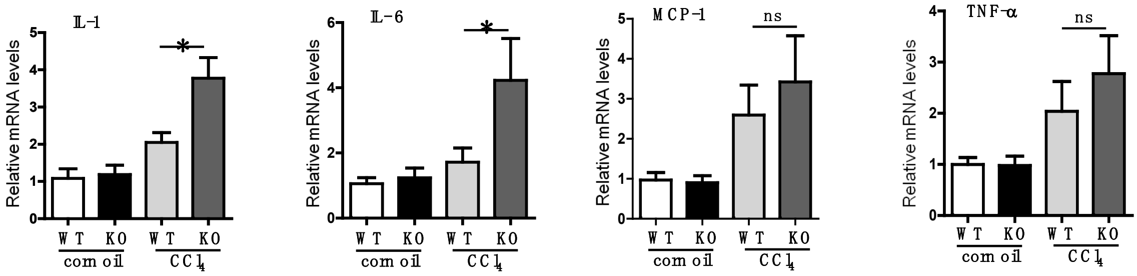

Fig.S4: P16 knockout (KO) and wild type (WT) mice were injected with CCl_4 as described in Methods. Expression levels of pro-inflammatory cytokines were examined by qPCR.

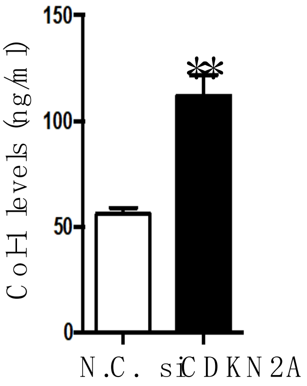

Fig.S5: LX-2 cells were transfected with siRNA targeting p16 or scrambled siRNA (SCR). Secreted collagen levels were examined with a Col 1 ELISA Kit according to the manufacturer's instruction (CUSABIO, China).

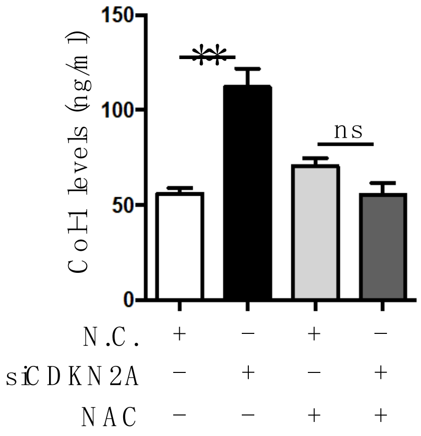

Fig.S6: LX-2 cells were transfected with siRNA targeting p16 or scrambled siRNA (SCR) followed by treatment with NAC. Secreted collagen levels were examined with a Col 1 ELISA Kit according to the manufacturer's instruction (CUSABIO, China).

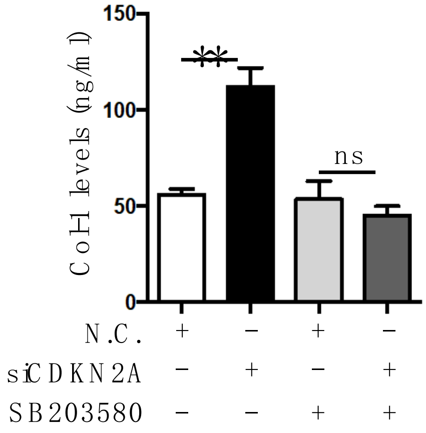

Fig.S7: LX-2 cells were transfected with siRNA targeting p16 or scrambled siRNA (SCR) followed by treatment with SB203580. Secreted collagen levels were examined with a Col 1 ELISA Kit according to the manufacturer's instruction (CUSABIO, China).
